# Dr. William Thomas Coggin

**Published:** 1894-08

**Authors:** 


### DR. WILLIAM THOMAS COGGIN.

It will be remembered that some months ago a claim was made through the medical press in behalf of Dr. W. T. Coggin, Athens, Ga., for the honor of having done the first American symphyseotomy. Dr. Coggin then alleged that while a resident of Keener, Ala., he operated upon a Mrs. Cary Hughs, the wife of a miner in Freedman in Northeast Alabama, delivering a living child by the pubic section; and that Dr. C. J. Slaughter assisted him in the operation.

*Tri-State News.

Some inquiring minds in the Etowah (Ala.) County Medical Society proceeded to investigate Dr. Coggin's claims, as well as the doctor himself. These results, as published in the *Alabama Medical and Surgical Age* (June), make some lively reading.

It now appears that there is no such place as Freedman, Ala.; that no such person as Cary Hughs was ever heard of in the alleged locality; and Dr. Slaughter says that he never assisted in any such operation by Dr. Coggin or anybody else.

In May the above medical society invited and urged Dr. Coggin to attend their June meeting and exhibit his patient and such other evidence as he might have to establish his claim. He failed to appear.

We pass over the damaging exposures concerning the doctor's private record. The publication referred to brands Dr. Coggin's claims as absolutely false, and the society challenges the doctor to contradict their statements. Dr. Coggin has written a letter to the *Alabama Medical and Surgical Age,* explaining mainly some personal differences between himself and some members of the above society. The chief matter at issue was not discussed, and therefore the society's position must stand.

